# CovidPubGraph: A FAIR Knowledge Graph of COVID-19 Publications

**DOI:** 10.1038/s41597-022-01298-2

**Published:** 2022-07-08

**Authors:** Svetlana Pestryakova, Daniel Vollmers, Mohamed Ahmed Sherif, Stefan Heindorf, Muhammad Saleem, Diego Moussallem, Axel-Cyrille Ngonga Ngomo

**Affiliations:** DICE Research Group, Department of Computer Science, Paderborn University, Paderborn, Germany

**Keywords:** Medical research, Diseases, Research data

## Abstract

The rapid generation of large amounts of information about the coronavirus SARS-CoV-2 and the disease COVID-19 makes it increasingly difficult to gain a comprehensive overview of current insights related to the disease. With this work, we aim to support the rapid access to a comprehensive data source on COVID-19 targeted especially at researchers. Our knowledge graph, CovidPubGraph, an RDF knowledge graph of scientific publications, abides by the Linked Data and FAIR principles. The base dataset for the extraction is CORD-19, a dataset of COVID-19-related publications, which is updated regularly. Consequently, CovidPubGraph is updated biweekly. Our generation pipeline applies named entity recognition, entity linking and link discovery approaches to the original data. The current version of CovidPubGraph contains 268,108,670 triples and is linked to 9 other datasets by over 1 million links. In our use case studies, we demonstrate the usefulness of our knowledge graph for different applications. CovidPubGraph is publicly available under the *Creative Commons Attribution 4.0 International* license.

## Abstract (summary)

**Table:** 

Measurement(s)	COVID-19-related publications
Technology Type(s)	named entity recognition, entity linking and link discovery approaches

## Background & Summary

The number of papers pertaining to SARS-CoV-2 and COVID-19 has surged over the last few months, making it hard to keep track of the latest research findings on the subject matter. Hence, the Allen Institute initiated a growing corpus of publications about COVID-19 called CORD-19^1^, which is updated on a regular basis. While the CORD-19 dataset provides the extracted full texts and corresponding licenses, it is still difficult to consume for end users and applications. For example, the data is available as one download (see https://www.semanticscholar.org/cord19/download). Hence, users first need to download the dataset and carry out some processing (e.g., some form of information retrieval) to get the information they desire. The integration of insights from different sources, which is of central importance in scientific research, cannot be carried out on the dataset directly. Moreover, the data being available in textual form makes it difficult to query using a structured query language such as SQL or SPARQL.

A growing number of research labs are hence building upon CORD-19 to make the data more amenable to automated processing. Table 1 gives an overview of existing datasets pertaining to COVID-19. Some datasets such as Wikidata Scholia only contain a small subset of the publications available as CORD-19. Other knowledge graphs about COVID-19 focus exclusively on case statistics instead of scientific publications (e.g., Covid-19 by STKO Lab) or text mining on the CORD-19 dataset without providing much information about the content present in the publications (e.g., Covid19-KG by Blender Lab and Cord-19-on-FHIR). Our goal differs from that of other COVID-19-related datasets: We aim to provide a comprehensive RDF representation of the CORD-19 data and include Natural Language Processing (NLP) results on the data to facilitate the development of intelligent search engines, domain-specific conversational AIs and structured machine learning solutions for COVID-19.

**Table 1:** Overview of COVID-19 datasets.

Dataset	Format	Endpoint	Publ.	Base
CovidPubGraph (DICE Lab) (publications, links to DrugBank, Sider, Kegg, Cord19-NEKG, LitCovid, …)	rdf	LodView	160,271	CORD-19^1^
Cord19-NEKG^9^ (Wimmics) (publications, links to DBpedia, Wikidata and BioPortal)	rdf	Virtuoso	111,256	CORD-19^1^
Covid-19-Literature^10^ (IDLab) (publications, links to DBpedia)	rdf	Download	40,750	CORD-19^1^
Wikidata Scholia^24^ (publications)	json/csv	WDQS		
Covid19-KG^12^ (Blender Lab) (genes, diseases, chemicals, organisms)	csv	Download	0	CORD-19^1^
Cord-19-on-FHIR^13^ (conditions, medications, procedures)	rdf	GraphDB	0	CORD-19^1^
Covid-19^21^ (STKO Lab) (case statistics by region)	rdf	GraphDB	0	JHU^30,31^

In this paper, we present CovidPubGraph, a comprehensive RDF knowledge graph of COVID-19 based on CORD-19. Our dataset follows the Linked Data lifecycle^2^. We provide a detailed representation of the COVID-19 publications in RDF including properties like publication title, authors names and their institutions, paper sections (e.g., abstract, introduction, body, discussion, etc.) and annotated references (e.g., references to figures). Resources such as authors and named entities augment the original data and make it easier to process for the sake of question answering and machine learning. All resources in the dataset are dereferenceable HTTP IRIs, which can be accessed via LodView (https://lodview.it/) or via the dataset’s SPARQL endpoint (https://covid-19ds.data.dice-research.org/sparql/). In addition, we link our dataset to the biomedical entities in other relevant datasets (e.g., DrugBank, Sider, Kegg).

Our knowledge graph also abides by the FAIR principles^3^: It is *findable* by virtue of being annotated with rich metadata and indexable by search engines. We make it *accessible* by providing our data via an RDF dump download (https://hobbitdata.informatik.uni-leipzig.de/COVID19DS/archive/), a SPARQL endpoint as well as dereferenceable individual resources. For example, see https://covid-19ds.data.dice-research.org/resource/4bf4b71883a26d15dcc13b2800ec470b99764956. We make it *interoperable* by employing standard vocabularies, e.g., for authors, papers, and sections within papers, as well as through the aforementioned links to 9 knowledge graphs including Cord19-NEKG, Cord-19-on-FHIR as well as Covid-19-Literature (see Table 2). We make it *reusable* by associating the data with clear provenance and licensing information as well as by reusing popular vocabularies such as NIF and Fabio ourselves.

**Table 2:** External datasets linking statistics.

Dataset	#Links	Predicate	Link classes
Cord19-NEKG^1^	160,271	owl:sameAs	Publications
LitCovid^2^	143,840	owl:sameAs	Publications
Covid-19-Literature^3^	160,271	owl:sameAs	Publications
Cord-19-on-FHIR^4^	160,271	owl:sameAs	Publications
Cord-19-on-FHIR	160,271	rdfs:seeAlso	Publications
Makg^5^	160,271	owl:sameAs	Publications
Makg	22,885	owl:sameAs	Authors
Makg	6,589	owl:sameAs	Institutions
Kegg^6^	202,482	itsrdf:taIdentRef	Named entities
Sider^7^	41,741	itsrdf:taIdentRef	Named entities
DrugBank^8^	78,969	itsrdf:taIdentRef	Named entities
Total number of links	1,297,861		

^1^http://ns.inria.fr/covid19/.

^2^http://ns.inria.fr/covid19/.

^3^https://www.ncbi.nlm.nih.gov/pmc/articles/.

^4^https://www.ncbi.nlm.nih.gov/pmc/articles/.

^5^https://data.linkeddatafragments.org/covid19.

^6^https://data.linkeddatafragments.org/covid19.

^7^https://fhircat.org/cord-19/fhir/.

^8^https://fhircat.org/cord-19/fhir/.

Potential use cases of our knowledge graph include:

- Finding papers about certain biomedical entities, e.g., drugs, side effects, genes, or proteins.
- Discovering links between specific genome subsequences and drugs.
- Training explainable machine learning models by running structured machine learning on selected named entities (e.g., drug names) to find similar drugs for clinical trials. The models can be trained with DL-Learner^4^, EvoLearner^5^, or DRILL^6^ and they learn class expressions in description logics based on the publication graph (e.g., drugs investigated by similar authors or in similar articles). The class expressions are comprehensible by domain experts.
- Supporting scientometric research on various aspects related to COVID-19 publications, such as international collaboration trends^7^ and peer review trends^8^, which would be informative for policy-makers and the scientific community.

## Methods

Knowledge graphs on the field of COVID-19 can be divided by their topics covered: publications, biomedical entities, and case statistics.

### Knowledge graphs of publications

Most knowledge graphs of COVID-19 publications are based on the COVID-19 Open Research Dataset (CORD-19) by the Allen Institute^1^. The CORD-19 dataset is based on papers and preprints from Semantic Scholar. Papers in CORD-19 are sourced from PubMedCentral (PMC), PubMed, the World Health Organization’s Covid-19 Database, and preprint servers bioRxiv, medRxiv, and arXiv^1^. While CORD-19 contains the full texts of scientific publications, it does not adhere to FAIR principles^3^, e.g., it is only available via download and does not use common vocabularies. The two knowledge graphs most closely related to ours are Cord19-NEKG^9^ and Covid-19-Literature^10^. However, neither of them provides comprehensive metadata about the publications, and neither provides fine-granular information pertaining to the publications (e.g., section information). An alternative to CORD-19 is the Lens dataset on COVID-19^11^. Lens contains metadata about scientific publications on COVID-19. However, it is only available as one big download (in JSON format). The Covidgraph project (https://covidgraph.org/) aims to utilize the dataset. However, at the time of writing, the proposed CovidPubGraph has not been released yet, making it hard to compare it to other knowledge graphs. To enable interoperability, we link our dataset to other datasets such as the Cord19-NEKG.

### Knowledge graphs of biomedical entities

Most works utilizing CORD-19 focus on extracting named entities^9,10,12,13^ such as genes, drugs, and proteins and linking them to existing knowledge bases such as DBpedia. For doing so, established tools such as DBpedia Spotlight^14^ and Entity Fishing (Wikidata) (https://github.com/kermitt2/entity-fishing/) are used. Alternatively, novel tools for recognizing biomedical entities on CORD-19 are also developed^15,16^. Noteworthy is also the work by *Zhou, Y. et al*.^17^, in which a network of genes, proteins, and viruses are proposed. The network is based on pre-existing biomedical databases (e.g., DrugBank, Therapeutic Target Database, and BindingDB) and does not cover the latest research findings. Still, such biomedical knowledge graphs might be employed to identify promising treatment options such as repurposing existing drugs or developing novel drugs regardless of the underlying construction methodology. We perform named entity recognition on CORD-19 and link the discovered entities to other biomedical RDF databases such as DrugBank^18^ (drugs), Sider^19^ (side effects), and Kegg^20^ (genes), thus making our dataset more amenable to tasks such as machine learning based on entities.

### Knowledge graphs of case statistics

Another class of knowledge graphs focuses on the case statistics of novel COVID-19 virus^21^, e.g., subdivided by region and based on the Dashboard data by the John Hopkins University.

## Data Records

### RDF data model design

The ontology behind our knowledge graph was derived from the source from which it was extracted, i.e., the full-texts of publications provided as part of the CORD-19 dataset. The ontology was designed to enable search, question answering and machine learning. At the time of writing, our dataset is based on CORD-19 version 2021-11-08 (https://www.semanticscholar.org/cord19/download). Our conversion process is implemented in *Python* 3.6 with *RDFLib* 5.0.0 (https://github.com/RDFLib/rdflib). We make our source code publicly available (https://github.com/dice-group/COVID19DS) to ensure the reproducibility of our results and the rapid conversion of novel CORD-19 versions. One version of the generated RDF dataset can be found at Zenodo^22^.

#### Listing 1.

List of all used vocabularies in CovidPubGraph.

% @prefix cvdr: https://covid-19ds.data.dice-research.org/resource/.

% @prefix cvdo: https://covid-19ds.data.dice-research.org/ontology/.

% @prefix bibo: http://purl.org/ontology/bibo/.

% @prefix bibtex: http://purl.org/net/nknouf/ns/bibtex#.

% @prefix dcterms: http://purl.org/dc/terms/.

% @prefix fabio: http://purl.org/spar/fabio/.

% @prefix foaf: http://xmlns.com/foaf/0.1/.

% @prefix its: http://www.w3.org/2005/11/its/rdf#.

% @prefix nif: http://persistence.uni-leipzig.org/nlp2rdf/ontologies/nif-core#.

% @prefix prov: http://www.w3.org/ns/prov#.

% @prefix rdf: http://www.w3.org/1999/02/22-rdf-syntax-ns#.

% @prefix rdfs: http://www.w3.org/2000/01/rdf-schema#.

% @prefix schema: http://schema.org/.

% @prefix sdo: http://salt.semanticauthoring.org/ontologies/sdo#.

% @prefix swc: http://data.semanticweb.org/ns/swc/ontology#.

% @prefix vcard: http://www.w3.org/2006/vcard/ns#.

% @prefix xml: http://www.w3.org/XML/1998/namespace.

% @prefix xsd: http://www.w3.org/2001/XMLSchema#.

% @prefix inria: http://ns.inria.fr/covid19/.

% @prefix ncbi: https://www.ncbi.nlm.nih.gov/pmc/articles/.

% @prefix pubnt: http://pubannotation.org/docs/sourcedb/CORD-19/sourceid/.

% @prefix ldf: https://data.linkeddatafragments.org/.

% @prefix fccc: https://fhircat.org/cord-19/fhir/Commercial/Composition/.

% @prefix makg: http://ma-graph.org/property/.

% @prefix dbo: https://dbpedia.org/ontology/.

#### RDF namespaces

To facilitate the reusability of our knowledge graph, we represent our data in widely used vocabularies and namespaces as shown in Listing 1.

#### RDF data model

**Fig. 1:**
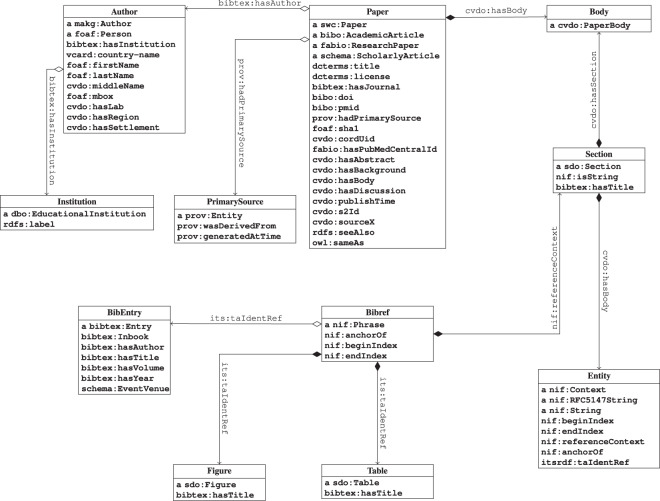
UML class diagram of the CovidPubGraph Ontology.

Figure 1 shows important classes (e.g., papers, authors, sections, bibliographic entries, and named entities) as well as predicates (e.g., first name, last name, license).

### Papers

We represent bibliographic information of papers using four vocabularies: bibo, bibtex, fabio, and schema (see namespaces above). Important attributes include the title, PMID, DOI, publication date, publisher, publisher URI, license and authors. For each paper, we store provenance information. In particular, our code allows the reference to the original CORD-19 raw files as well as the time when we generate the resource. The URIs of our generated Paper resources follow the format https://covid-19ds.data.dice-research.org/resource/<paperId> where <paperId> is the unique paper id within the CORD-19 dataset. An example resource is given in Listing 2.

### Authors

Authors are represented in FOAF (http://xmlns.com/foaf/spec/). Important attributes include the first, middle, and last names as well as mail addresses and institutions.

### Sections

Papers are further subdivided by section and the corresponding information is expressed in the SALT ontology^23^. We keep track of a set of predefined sections including Abstract, Introduction, Background, Related Work, Preliminaries, Conclusion, Experiment and Discussion. In case another section heading appears in the paper, we assign it to the default section Body. We further subdivide a section using cvdo:hasSection. An example is given in Listing 3.

### References

References to other sections, figures and tables in the text are resolved and stored as RDF using Bibref. Important attributes are the anchor of the reference (e.g., the number of the section, figure, or table), its source string in the text (nif:referenceContext) along with its position in the text (nif:beginIndex, nif:endIndex) as well as the referenced object (its:taIdentRef) which might be a paper (BibEntry), a figure (Figure), or a table (Table).

#### Listing 2.

Example paper resource.

### Named entities

As machine learning and question answering often rely on named entities and their locations in texts, we annotate CORD-19 papers accordingly and represent this information with the NIF 2.0 Core Ontology (https://persistence.uni-leipzig.org/nlp2rdf/ontologies/nif-core/nif-core.html). Further details of our entity linking process are described in Linking Section.

### RDF example resources

Listing 2 provides an example of a paper represented as an RDF resource. Listing 3 shows an example of a section resource. Each section is linked to its text string via nif:isString and its title via bibtex:hasTitle. If a section includes references to other papers, figures or tables (e.g., (1-3), (4,5), Figure 1A,Fig. 1, etc.), we represent a reference in RDF as follows: We represent the anchor of the reference with nif:anchorOf (e.g., the number of a figure), the start position of the reference with nif:beginIndex, the end position of the reference with nif:endIndex, the source section of the reference with nif:referenceContext, and the referenced target with its:taIdentRef (e.g., a bibtex entry, figure or table). An example is shown in Listing 4. Listing 5 shows an example of provenance information.

### Linking

We link our dataset to other data sources to ensure its reusability and integrability as well as to improve its use for search, question answering and structured machine learning. We generate links from our paper and author resources to publicly available related knowledge bases. Moreover, we extract named entities related to diseases, genes, and cells from all converted papers and link them to three external knowledge bases.

#### Linking publications, authors and institutes

We link *publications* in our knowledge graph to six other datasets using the owl:sameAs and rdfs:seeAlso predicates (see top six rows of Table 2). To the best of our knowledge, those six datasets are the most relevant RDF datasets that deal with the same publication data. We leave it to future work to link our dataset to non-RDF datasets such as Covid19-KG^12^ and Wikidata Scholia^24^.

##### **Listing 3.**

Example section representation.

##### **Listing 4.**

Example of a reference to a paper and its associated bibtex entry.

Cord19-NEKG and our dataset use the same CORD-19 paperId making the linking process straightforward. For LitCovid, we use the PubMed Central Id (PMC-id) that is provided as part of CORD-19. For Covid-19-Literature and Cord-19-on-FHIR, we employ sha hash values from CORD-19. Moreover, we link our dataset to the publications’ JSON files in Cord-19-on-FHIR with the predicate rdfs:seeAlso. Listing 6 shows an example of linked publications from our dataset CovidPubGraph to Cord19-NEKG and LitCovid.

We link our resources of both our *authors* and *institutes* to the *Microsoft Academic Knowledge Graph* (MAKG)^25^ using the latest version of our link discovery framework LIMES^26^. For linking the *authors*, LIMES is configured to discover owl:sameAs links between our instances of foaf:Person and Microsoft’s makg:Author. For linking the *institutes*, we look for links between instances of type dbo:EducationalInstitution from our knowledge graph and MAKG’s resources of type makg:Affiliation. LIMES configuration files for linking authors and institutes are available as part of our source code (https://github.com/dice-group/COVID19DS).

#### Linking named entities

We apply entity linking to connect entities derived from the sections of papers to other knowledge bases. This process comprises two steps: (1) entity extraction and (2) entity linking. For the extraction step, we use Scispacy^27^ in version 0.2.4 in conjunction with the model en_ner_bionlp13cg_md (https://github.com/allenai/scispacy) which allows the extraction of biomedical entities such as diseases, genes and cells. Scispacy is a specialized NLP library based on the spaCy library (https://spacy.io/). The NER model in spaCy is a transition-based chunking model that represents tokens as hashed embedded representations of the prefix, suffix, shape and lemmatized features of individual words^27^.

##### **Listing 5.**

Provenance information for the non-commercial dataset.

##### **Listing 6.**

An example of a linked publication.

##### **Listing 7.**

Entity linking example.

For the linking step, we adapt the entity linking framework MAG^28^ to link our extracted resources to the three knowledge bases Sider^19^, Kegg^20^ and DrugBank^18^—using their RDF versions provided by the Bio2RDF project (https://bio2rdf.org/). We adapt MAG by creating a search index for each of the external knowledge bases and running MAG once per knowledge base. The output is a set of entities in the NLP Interchange Format (NIF) (https://persistence.uni-leipzig.org/nlp2rdf/). In Listing 7, we provide an example for the named entity *“folic acid”*.

### Automated generation of CovidPubGraph

**Fig. 2:**
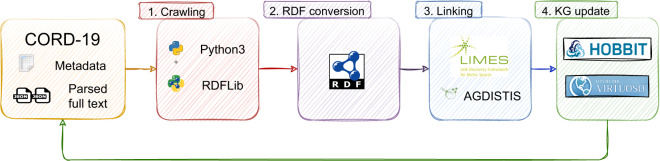
CovidPubGraph pipeline.

CORD-19 uploaded new data almost every day for the second half of 2020. Due to this fact, we have to automate the process of updating our knowledge graph. To this end, we developed a pipeline to automate the entire process, which can be found in Fig. 2. This pipeline contains several steps:

1. **Crawling**. We start by crawling the most recent version as a zip file from the CORD-19 website, which includes a CSV metadata file and JSON parsed full texts of scientific papers about the coronavirus.
2. **RDF conversion**. Then, we convert the CORD-19 data into an RDF knowledge graph with a Python script using the RDFLib library (https://github.com/RDFLib/rdflib).
3. **Linking**. We integrate the AGDISTIS library (https://github.com/dice-group/AGDISTIS) into the generation process to extract and link the named entities from abstracts of the scholarly articles. Moreover, we carry out the entity linking tasks (i.e., link *publication* and *authors* to other datasets) by making use of the link discovery framework LIMES (https://github.com/dice-group/LIMES).
4. **KG Update**. We upload the new version of CovidPubGraph dumps into the HOBBIT server (https://hobbitdata.informatik.uni-leipzig.de/COVID19DS/archive/) as well as to the Virtuoso triple store (https://hub.docker.com/r/openlink/virtuoso-opensource-7).

Starting from 2021, CORD-19 publishes new data only every two weeks. Therefore, we keep our KG up-to-date by crawling the new version of the CORD-19 dataset biweekly. Then, we follow the KG creation procedure presented in Fig. 2. As the dataset is still not too big to be regenerated, we regenerate the complete dataset biweekly. Still, having an automatic incremental update is part of our future plans.

## Technical Validation

Representing COVID-19-related publications as RDF promises to facilitate many applications and use cases—some of which we outline in this section.

### Updating the dataset

An example of how the data are constantly updated is provided in Table 3, where we provide details about the growing number of different resource types across successive versions of our knowledge graph. As we trust the data provider, i.e. the Allen Institute, we do not do any further data cleaning than the pipeline introduced in Fig. 2. Moreover, the number of generated links to other external datasets within our linking (see Table 2), provides further evidence of the quality of the data.

**Table 3:** CovidPubGraph statistics.

	Version 1.0	Version 2.0	Version 27.0	Version 28.0
Distinct number of over all resources	11,249,740	15,761,537	214,036,877	268,108,670
Distinct number of publications	40,224	58,739	216,664	262,954
Distinct number of authors	1,434,809	1,484,024	2,892,156	3,388,001
Distinct number of bib entries	1,482,257	2,022,147	6,156,150	7,748,575
Distinct number of bib figures	333,509	461,386	1,243,561	1,532,443
Distinct number of bib tables	158,896	251,970	538,523	690,478

#### Listing 8.

List the top 10 papers-URIs with the most number of authors.

#### Listing 9.

List all paper URIs written by the author “Ian Mackay.”

### Data retrieval

While our base dataset CORD-19 contains a significant number of publications, they are not represented in a format optimized for retrieval.

By providing CovidPubGraph in RDF with a well-defined ontology, we enable the easy retrieval of data with structured query languages such as SPARQL. For example, Listing 9 shows a query to retrieve all papers written by the author “Ian Mackay.” Another query to retrieve the top 10 papers in terms of their number of authors is provided in

Using SPARQL queries, we carried out some random checks of the duplicate articles and authors, which resulted in no duplicates. This could be a direct consequence of the high quality of the original CORD dataset. Still, doing a full KG deduplication task is part of our future work.

### Interoperability using NIF

Using the interoperability capabilities provided by NIF, it is easy to query all occurrences of a certain text segment within the whole dataset and still know exactly where each mention occurs. For example, in Listing 10, we provide a SPARQL query to list all papers where “folic acid” is mentioned with their respective sections.

### Information aggregation

Linking our dataset to other RDF datasets adds a considerable amount of value. For example, Microsoft Academic Knowledge Graph (MAKG) covers more than 209 million publications (http://ma-graph.org/) and our interlinking enables the retrieval of an author’s citation count (Listing 11).

## Usage Notes

Table 4 summarizes all technical details of our dataset pertaining to its availability.

**Table 4:** Technical details of CovidPubGraph.

Name	CovidPubGraph
**Example Resource**	https://covid-19ds.data.dice-research.org/resource/pmc4913562
**Dataset dump**	https://hobbitdata.informatik.uni-leipzig.de/COVID19DS/archive/
**Archived Dump**	10.5281/zenodo.4650261
**Sparql Endpoint**	https://covid-19ds.data.dice-research.org/sparql
**Dataset Graph**	https://covid-19ds.data.dice-research.org/resource/corona
**Ontology**	https://covid-19ds.data.dice-research.org/ontology/
**Void File**	https://covid-19ds.data.dice-research.org/void/
**Ver. Date**	November 8, 2021
**Ver. No**.	28.0
**Source Code**	https://github.com/dice-group/COVID19DS
**Software License**	GPL 3.0 (https://www.gnu.org/licenses/gpl-3.0)
**Dataset License**	Creative Commons Attribution 4.0 International (https://creativecommons.org/licenses/by/4.0/)

### Persistent URIs

All our resources are served from one of our servers via persistent URIs. The resource will be maintained by the DICE research team (https://dice-research.org) as part of the lab’s HOBBIT dataset efforts^29^. A 100TB-Server maintained by the Paderborn university’s computing centre will host the datasets.

### Resource dereferencing

**Fig. 3:**
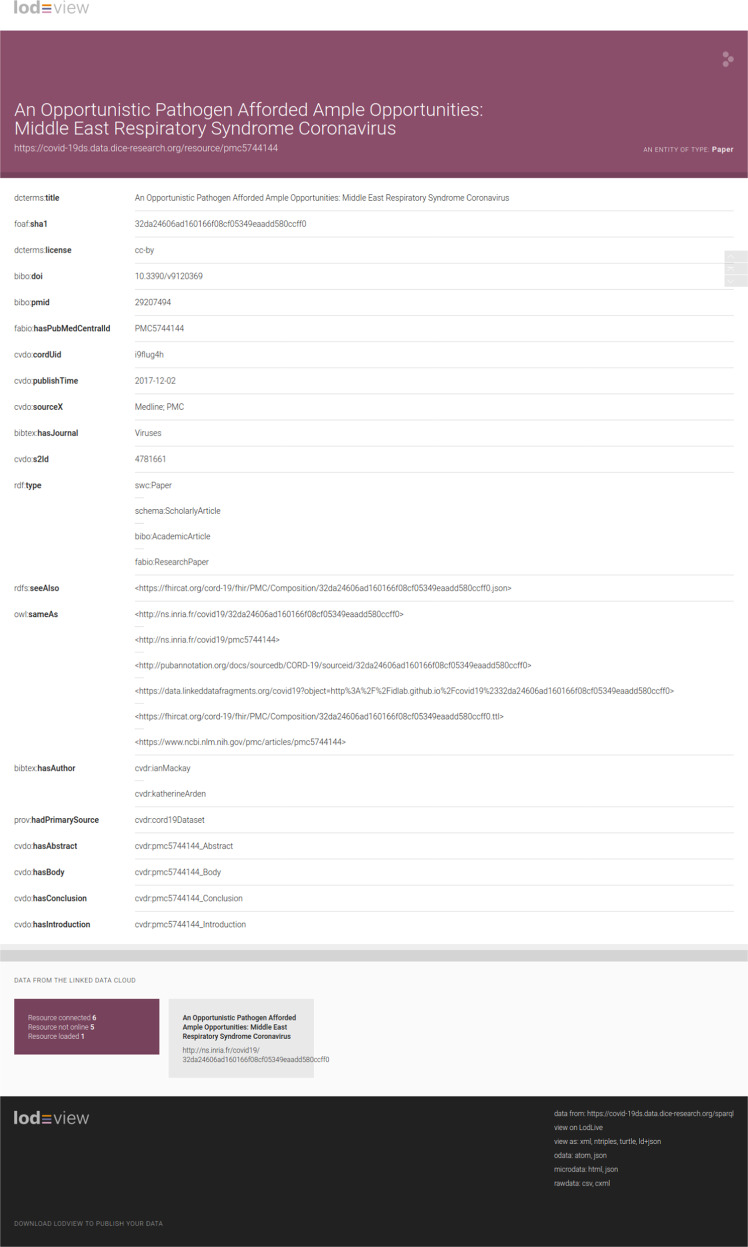
Excerpt of an example resource served by LodView.

We employ LodView (https://lodview.it/) for dereferencing our dataset URIs and allowing users to conveniently browse HTML pages. Figure 3 shows an example of a resource being served by LodView.

#### Listing 10.

List all papers and sections mentioning “folic acid.”

#### Listing 11.

SPARQL example for retieving more data via interlinking with MAKG.

### Dump files

We provide dump files of our dataset for download. The generated RDF datasets are located on our HOBBIT storage (https://hobbitdata.informatik.uni-leipzig.de/COVID19DS/archive/) and archived on Zenodo (https://zenodo.org/record/4650261).

### SPARQL endpoint

We publicly serve CovidPubGraph via a SPARQL endpoint (https://covid-19ds.data.dice-research.org/sparql).

## Data Availability

Our source code to generate the new versions of our knowledge graph is publicly available at https://github.com/dice-group/COVID19DS and is maintained in parallel with the knowledge graph.
